# Use of Cannabis for Sleep: Patterns of Use Among a Sample of Patients in a Geriatrics Clinic

**DOI:** 10.1093/geroni/igaa057.1927

**Published:** 2020-12-16

**Authors:** Christopher Kaufmann, Kevin Yang, Atul Malhotra, Khai Nguyen, Reva Nafsu, Alison Moore

**Affiliations:** 1 University of California San Diego, San Diego, California, United States; 2 University of California San Diego, La Jolla, California, United States

## Abstract

Cannabis may be useful for treatment of sleep problems in older adults. Little is known about use patterns of cannabis for sleep. We conducted a clinic-based anonymous survey in a geriatrics clinic in La Jolla, CA. Among n=568 surveys, 10% reported recent use (within past 6 months). Among recent users, 30% reported using cannabis for sleep, most finding it helpful. Compared to cannabis users for other conditions, cannabis users for sleep were more likely to use THC containing products (62.5% vs. 32.1%), use cannabis on a daily or weekly basis (76.2% vs. 43.2%), use vape pens (29.2% vs. 6.9%), and to obtain cannabis via a delivery service (33.3% vs. 14.0%) (all p’s<0.05). Only 40% reported their doctor knew about their cannabis use. Our findings suggest sleep is a common reason for using cannabis. Future research should assess how use patterns can result in effective treatment for sleep in older adults. Part of a symposium sponsored by the Sleep, Circadian Rhythms and Aging Interest Group.

